# Follicular Helper T Cells in DiGeorge Syndrome

**DOI:** 10.3389/fimmu.2018.01730

**Published:** 2018-07-23

**Authors:** Adam Klocperk, Zuzana Paračková, Markéta Bloomfield, Michal Rataj, Jan Pokorný, Susanne Unger, Klaus Warnatz, Anna Šedivá

**Affiliations:** ^1^Department of Immunology, 2nd Faculty of Medicine, Charles University in Prague and Motol University Hospital, Prague, Czechia; ^2^Center for Chronic Immunodeficiency (CCI), Medical Center-University of Freiburg, Faculty of Medicine, Freiburg im Breisgau, Germany; ^3^Department of Rehabilitation and Sports Medicine, 2nd Faculty of Medicine, Charles University in Prague and Motol University Hospital, Prague, Czechia

**Keywords:** immunodeficiency, DiGeorge, T cells, follicular helper T cells, PD1, ICOS, memory, thymus

## Abstract

DiGeorge syndrome is an immunodeficiency characterized by thymic dysplasia resulting in T cell lymphopenia. Most patients suffer from increased susceptibility to infections and heightened prevalence of autoimmune disorders, such as autoimmune thrombocytopenia. B cells in DiGeorge syndrome show impaired maturation, with low switched-memory B cells and a wide spectrum of antibody deficiencies or dysgammaglobulinemia, presumably due to impaired germinal center responses. We set out to evaluate circulating follicular helper T cells (cTFHs) in DiGeorge syndrome, as markers of T–B interaction in the germinal centers in a cohort of 17 patients with partial DiGeorge and 21 healthy controls of similar age. cTFHs were characterized as CXCR5^+^CD45RA^−^ CD4^+^ T cells using flow cytometry. We verify previous findings that the population of memory CD4^+^ T cells is relatively increased in diGeorge patients, corresponding to low naïve T cells and impaired T cell production in the thymus. The population of CXCR5^+^ memory CD4^+^ T cells (cTFHs) was significantly expanded in patients with DiGeorge syndrome, but only healthy controls and not DiGeorge syndrome patients showed gradual increase of CXCR5 expression on cTFHs with age. We did not observe correlation between cTFHs and serum IgG levels or population of switched memory B cells. There was no difference in cTFH numbers between DiGeorge patients with/without thrombocytopenia and with/without allergy. Interestingly, we show strong decline of PD1 expression on cTFHs in the first 5 years of life in DiGeorge patients and healthy controls, and gradual increase of PD1 and ICOS expression on CD4^−^ T cells in healthy controls later in life. Thus, here, we show that patients with DiGeorge syndrome have elevated numbers of cTFHs, which, however, do not correlate with autoimmunity, allergy, or production of immunoglobulins. This relative expansion of cTFH cells may be a result of impaired T cell development in patients with thymic dysplasia.

## Introduction

DiGeorge syndrome is a primary immunodeficiency characterized by thymic dysplasia and T-cell lymphopenia (1). Its most common cause is a 3 Mb deletion on the 22nd chromosome (del22q11.2), which among others encompasses also the *TBX* gene, responsible for the formation of thymic anlage, a basic structural foundation of the thymus, and its further fetal development (2). This failure to develop a proper niche for the generation of mature thymocytes results in T-cell lymphopenia and increased susceptibility to infection in patients with DiGeorge syndrome. Other clinical symptoms of this syndrome include congenital heart disease, hypoparathyroidism, developmental retardation, and an increased prevalence of autoimmune disease (3–7).

The immune system has been studied thoroughly in DiGeorge syndrome, with a specific focus on T cells and their development. While only 1.5% of patients present with complete DiGeorge syndrome and suffer from life-threatening severe T-cell lymphopenia (8), even patients with partial DiGeorge syndrome show T-cell lymphopenia and decrease of thymic output with low naïve T cells, recent thymic emigrant T cells reflected by low number of T-cell receptor excission circles (4, 9, 10). The impaired T-cell development is further shown to cause oligoclonality within the T-cell compartment (11). Taken together with information on the humoral immune compartment in DiGeorge syndrome patients, including impaired response to vaccination, hypogammaglobulinemia (12, 13), and dysfunctional maturation of B-cells (4, 14, 15), these findings reflect the dysregulation of T–B-cell interactions in DiGeorge syndrome.

The principal subset of T cells crucial for the proper development of germinal center response, B-cell class-switching, and establishment of humoral memory are the follicular helper T cells. These cells are characterized by expression of chemokine receptor CXCR5, which allows their homing along the CXCL13 chemokine gradient produced mainly by follicular dendritic cells in germinal centers (16), thus ensuring their temporospatial colocalization with naïve B cells during the germinal center response to antigen. TFH cells produce IL-21 and express B-cell costimulatory molecules such as ICOSL, CD40L, and others, which promote B-cell proliferation, affinity maturation, and class-switching (17). While TFHs are mostly present in the secondary lymphoid organs, the peripheral blood contains a small population of cells that are generally accepted to be the circulating counterparts of TFH cells [thus circulating follicular helper T cells (cTFHs)] (18, 19). Numerous phenotypic characteristics have been proposed and used, generally including memory marker CD45RO or the absence of CD45RA, the chemokine receptors CXCR5, CCR6, CXCR3, activation/costimulation molecules PD1 and ICOS or the transcription factor Bcl-6. Similarly to changing proportions of naïve vs memory and other T-cell subsets during an individual’s life (20), the amount and quality of cTFHs is likely to change over time and has already been shown to decrease in the elderly (21).

There have been several reports describing cTFHs in various primary immunodeficiencies (22–24), but limited information is available on cTFHs in patients with DiGeorge syndrome, even though the combination of dysregulated T-cell development, impsaired humoral immunity, and immune dysregulation makes this syndrome a prime candidate for evaluation of cTFH cells. An earlier report by Derfalvi et al. found increased percentage of CXCR5^+^ICOS^+^ CD4 T cells in DiGeorge syndrome patients both below 17 years of age and adults (25). However, no further age-specific resolution was provided or clinical correlation discussed. We, therefore, investigated the cTFH population in pediatric patients with partial DiGeorge syndrome.

## Materials and Methods

### Patients

We present the results of 17 patients with partial DiGeorge syndrome (age 0.5–21 years, mean 7.6 years, 12 females, 5 males), compared to 21 healthy controls (age 0.1–22 years, mean 11.6 years, 9 females, 12 males). Basic patient data are summarized in Table 1. All of the patients harbor a del22q11.2 deletion verified through multicolor fluorescent *in situ* hybridization using the DiGeorge/VCFS TUPLE 1/22q Deletion Syndrome LPU004 probe (Cytocell, Cambridge, UK), and at the time of diagnosis, they fulfilled the ESID diagnostic criteria for DiGeorge syndrome. This study was carried out in accordance with the recommendations of the Ethical Committee of the second Faculty of Medicine, Charles University in Prague and University Hospital in Motol, Czech Republic. The protocol was approved by the Ethical Committee. All subjects gave written informed consent in accordance with the Declaration of Helsinki.

**Table 1:** Cohort characteristics.

Patient ID	Age (years)	Lymphocytes (×10^9^/l)	CD3 (% of lympho)	CD3 (×10^9^/l)	CD4 (% of lympho)	CD4 (×10^9^/l)	IgG (g/l)	IgM (g/l)	Thrombocytopenia	Allergy	SwM B cells (% of B cells)
Patient 1	0.2	8.32	34	2.83	12	1.00	2.47	0.30	No	No	NA
Patient 2	0.6	1.84	19	0.35	15	0.28	6.64	0.38	No	No	NA
Patient 3	1.6	5.32	67	3.56	28	1.49	9.56	0.59	No	No	NA
Patient 4	1.6	1.91	62	1.18	45	0.86	3.98	0.51	Yes	No	NA
Patient 5	3.5	2.10	53	1.11	34	0.71	12.6	0.28	No	No	8.4
Patient 6	4.5	NA	45	NA	23	NA	11.1	0.78	No	No	NA
Patient 7	5.2	3.49	54	1.88	34	1.19	7.55	0.22	No	No	11.7
Patient 8	5.3	2.67	51	1.36	27	0.72	11.2	0.59	No	No	6.6
Patient 9	5.5	2.55	72	1.84	43	1.10	9.77	0.83	No	Yes	NA
Patient 10	5.6	5.63	73	4.11	33	1.86	11.5	0.62	No	No	10.7
Patient 11	7.0	1.90	40	0.76	22	0.42	9.55	0.65	No	No	5.6
Patient 12	10.5	1.78	62	1.10	34	0.61	12.4	0.88	No	No	14.2
Patient 13	11.0	2.77	58	1.61	26	0.72	11.7	1.11	Yes	No	6.7
Patient 14	13.0	1.89	60	1.13	44	0.83	9.93	0.58	Yes	Yes	9.9
Patient 15	14.5	1.68	67	1.13	40	0.67	17.7	1.62	Yes	Yes	NA
Patient 16	19.5	1.57	71	1.11	43	0.68	10.1	0.41	No	Yes	1.6
Patient 17	20.5	2.01	61	1.23	45	0.90	20.2	3.03	Yes	No	13.3

*Table describing patients with DiGeorge syndrome included in this study, including basic laboratory and clinical data*.

### Flow Cytometry

**Figure 1:**
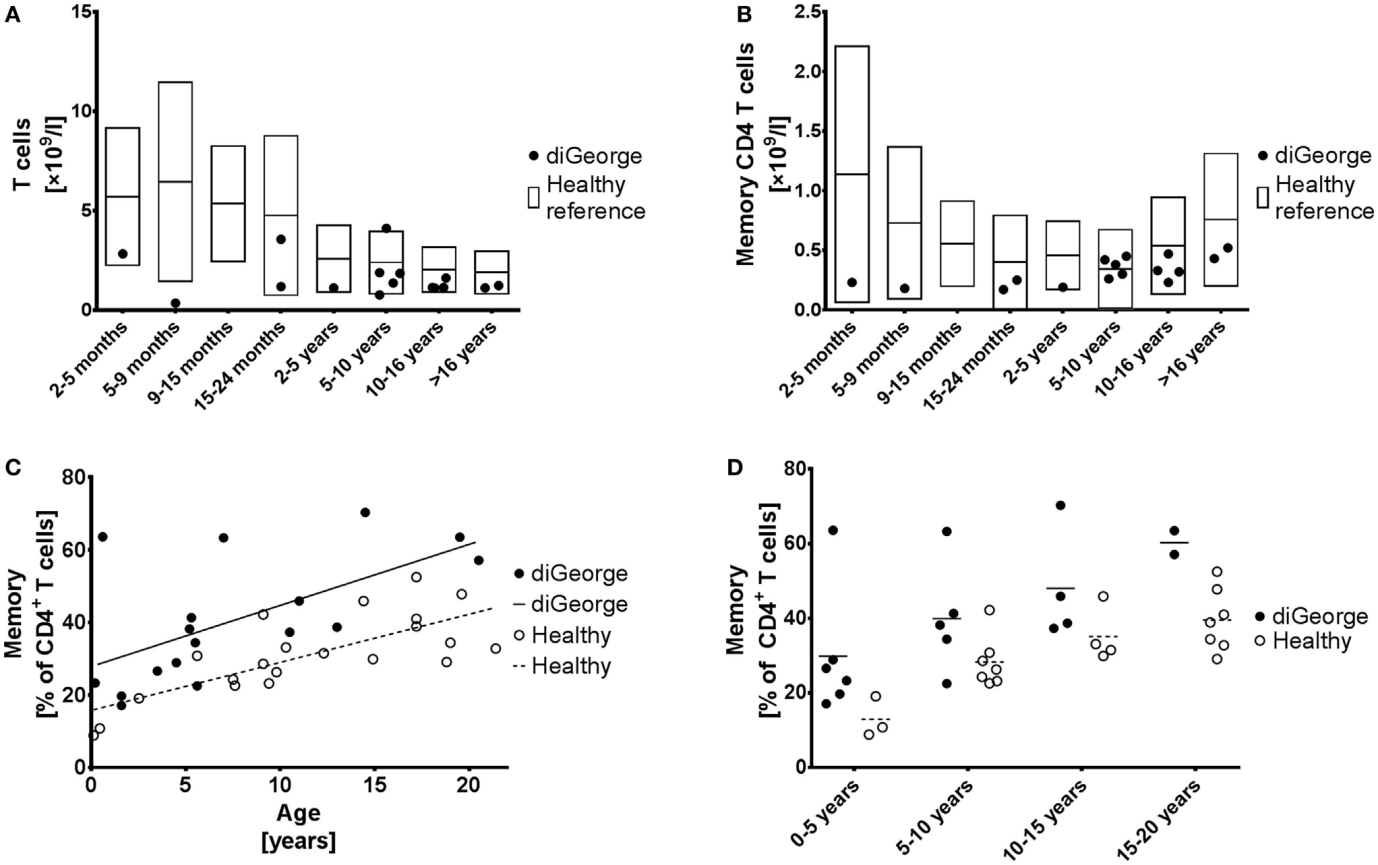
Memory CD4^+^ T cells. **(A)** Absolute T cell numbers and **(B)** absolute memory CD4^+^ T cell numbers in patients with DiGeorge syndrome, compared to published healthy reference values (20), shown as mean and 90% range. **(C)** Proportion of memory CD4^+^ T cells of all CD4^+^ T cells compared to age, shown with linear regression trendlines and **(D)** divided into several age groups.

Peripheral blood was taken as part of other routine investigations into EDTA-coated tubes, peripheral blood mononuclear cells were isolated using Ficoll-Paque gradient and stained with anti-CD3 Alexa Fluor 700 (clone MEM-57), anti-CD4 Pacific Blue (clone MEM-241, both from Exbio, Czech Republic), anti-CD45RA PE-Cy7 (clone HI100), anti-CXCR5 Alexa Fluor 488 (clone J252D4), anti-PD1 APC (clone EH12.2H7, all from BioLegend, San Diego, CA, USA), and anti-ICOS PE (clone ISA-3, ThermoFisher, MA, USA). Data were acquired on BD FACSAria II cytometer (BD Biosciences, USA) and analyzed using FlowJo VX (FlowJo, LLC, USA) and GraphPad Prism 6 (GraphPad Software, USA). Gating strategy is shown in Figure 1A.

## Results

### Patients with DiGeorge Syndrome Have High Memory CD4^+^ T-Cells due to Comparative Decrease of Naive CD4^+^ T-Cells

Our cohort of DiGeorge syndrome patients has typically low absolute T cell lymphopenia, which becomes less pronounced with age (Figure 1A). Reflecting this finding and we also observe low absolute memory CD4^+^ T cells (Figure 1B); however, the low thymic output of naïve T celly compartment (Figures 1C,D). This increase starts already at birth, remains constant throughout childhood s as shown by other groups (4, 9) results in relative increase of the memorand adolescence, and is highly significant (linear regression intercept *p* = 0.0002, slope *p* = 0.54) (Figure 1C).

### cTFHs Are Expanded in DiGeorge Syndrome

**Figure 2:**
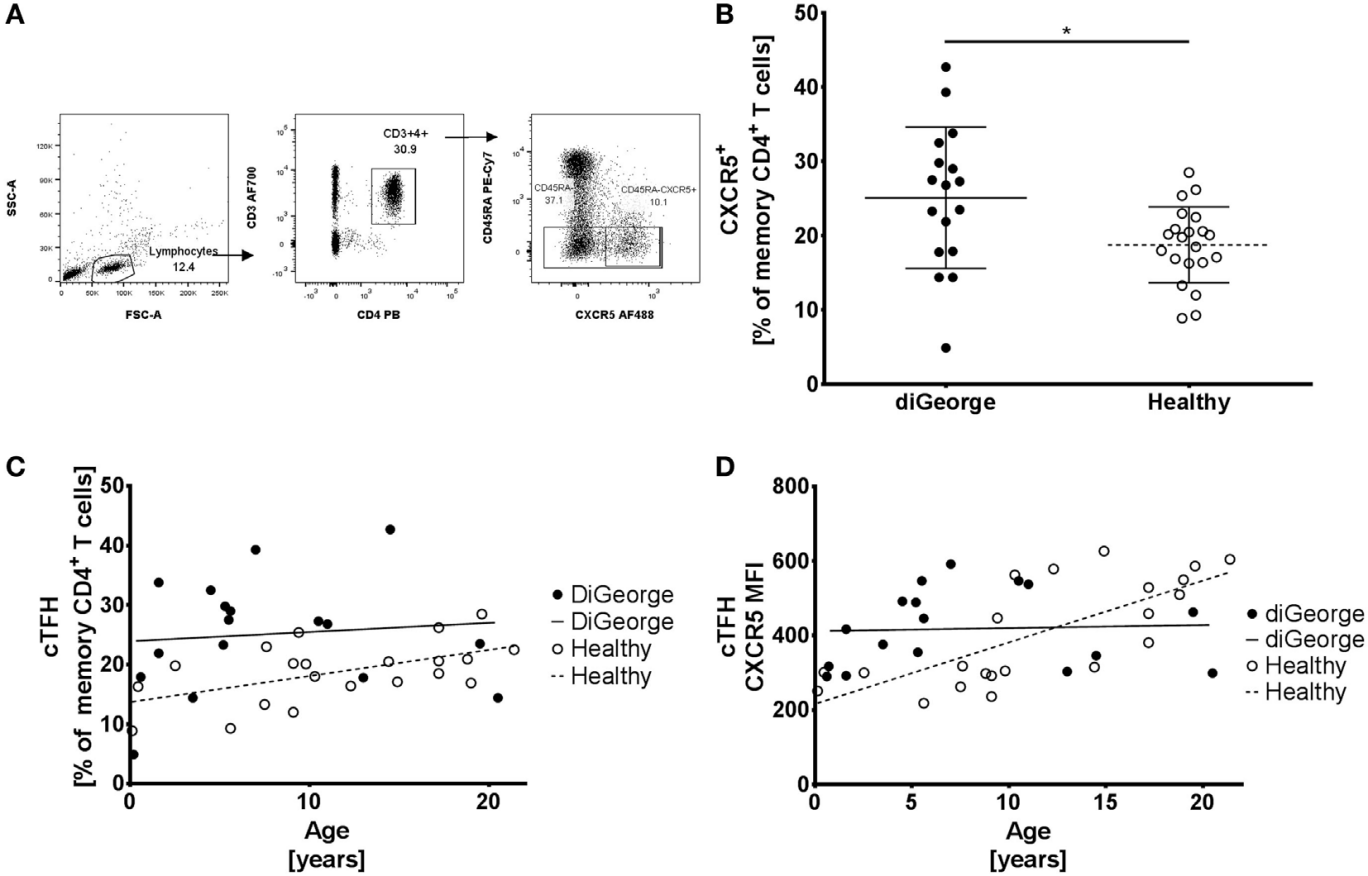
Gating strategy, circulating follicular helper T cells (cTFHs), and CXCR5 expression **(A)** Gating strategy of CD45RA^−^ (memory) CD4^+^ T cells and CD45RA^−^CXCR5^+^ (cTFH) CD4^+^ T cells. **(B)** Proportion of cTFH in memory CD4^+^ T cells in DiGeorge syndrome patients and healthy controls shown in summary (lines denote mean ± SD) and **(C)** as development over time with linear regression trendlines. **(D)** Expression of CXCR5 on cTFHs over time (lines denote linear regression trendlines).

To correct for the relative increase of memory CD4^+^ T cells in DiGeorge, we compared the percentages of CXCR5^+^ memory CD4^+^ T cells (cTFHs) of all memory CD4^+^ T cells (gating strategy shown in Figure 2A). We found that patients with DiGeorge syndrome have significantly elevated proportion of cTFH within the memory compartment compared to healthy controls (Welch’s *t*-test, *p* = 0.02) (Figure 2B).

However, whereas this proportion increased with age in healthy controls (linear regression, *p* = 0.01, *R*^2^ = 0.29) (Figure 2C), there was no significant increase of cTFH/memory CD4^+^ T cell proportion over time in DiGeorge patients (linear regression, *p* = 0.69, *R*^2^ = 0.01) (Figure 2C). This trend is further corroborated by gradual increase of CXCR5 expression on cTFHs in healthy patients (linear regression, *p* = 0.0001, *R*^2^ = 0.54) but not DiGeorge patients (linear regression, *p* = 0.68, *R*^2^ = 0.01) (Figure 2D).

### cTFH Are Not Markers of Humoral Immune Dysregulation in DiGeorge Syndrome

In order to evaluate whether cTFH population reflects the humoral immune dysregulation seen in patients with DiGeorge syndrome, we compared it to serum IgG levels, switched memory B cells, thrombocytopenia, and allergy.

**Figure 3:**
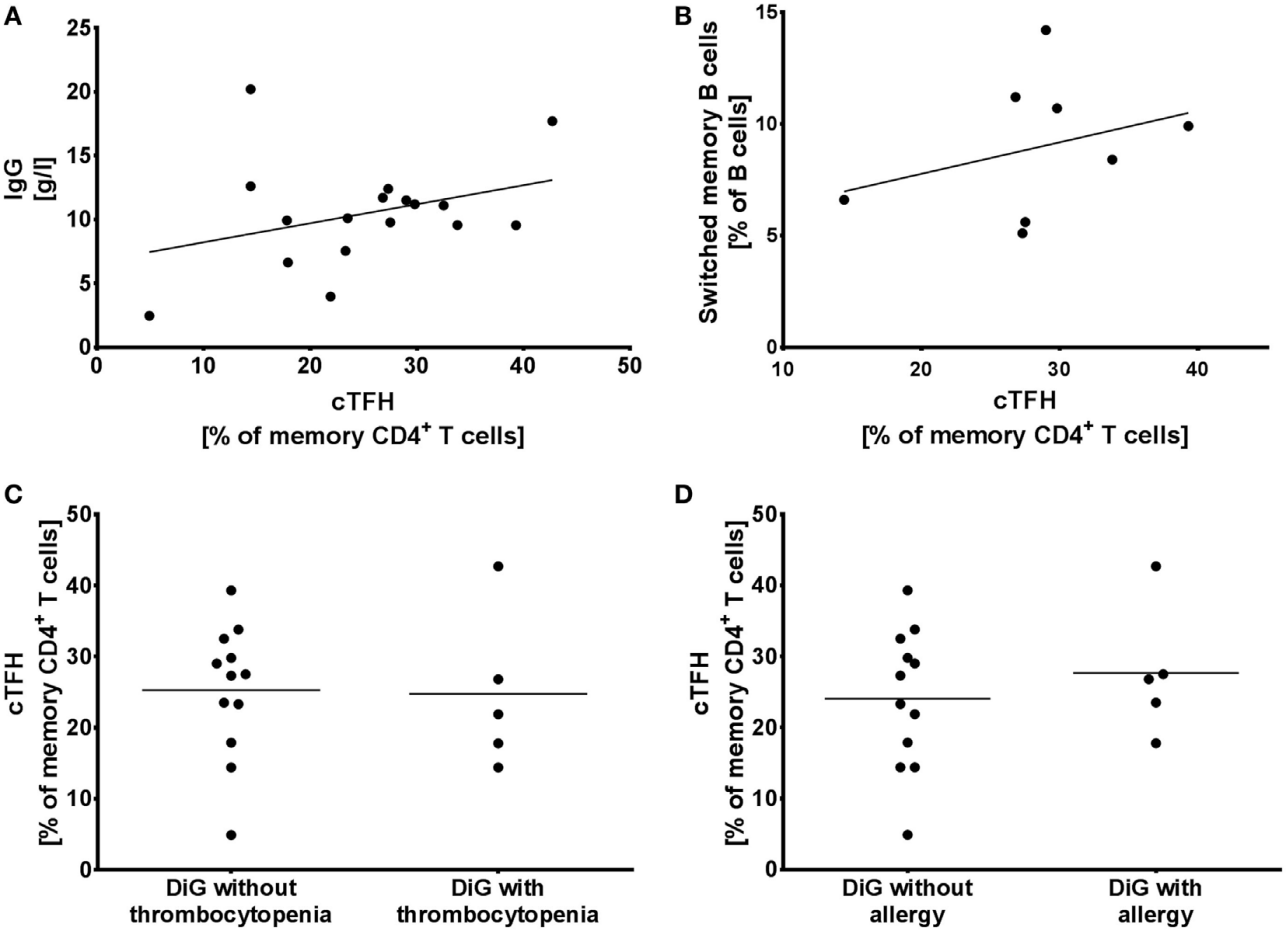
Circulating follicular helper T cells (cTFH) in humoral immune dysregulation, autoimmunity, and allergy. **(A)** Proportion of cTFHs in memory CD4^+^ T cells compared to serum IgG levels and **(B)** switched memory B cells in DiGeorge syndrome patients, shown with linear regression trendlines. **(C)** cTFHs in DiGeorge syndrome patients without/with thrombocytopenia and **(D)** without/with allergy (lines denote mean).

2/17 patients (12%) in our cohort suffered from hypogammaglobulinaemia (Patients 1 and 4), but hypergammaglobulinemia is seen in 7/17 patients (41%) (Patients 5, 6, 8, 10, 12, 15, and 17). However, we observed no correlation between the elevated cTFHs seen in Figure 2B and serum IgG levels (linear regression, *p* = 0.19, *R*^2^ = 0.11) (Figure 3A).

Similarly, despite the observed elevated cTFHs, there is a block in B cell maturation with low class-switched memory B cells in DiGeorge syndrome (11, 14). We compared cTFH numbers with switched memory B cells measured as part of previous investigations (0–2 years prior to evaluation of cTFH counts) (14), but saw no correlation (Figure 3B) (linear regression, *p* = 0.44, *R*^2^ = 0.10).

To investigate the influence of cTFHs on clinical phenotype of patients, we compared cTFH numbers in patients with/without thrombocytopenia (Figure 3C)—the most commonly seen autoimmune complication in DiGeorge syndrome—and with/without allergy (Figure 3D). There was no difference in cTFHs between these cohorts, suggesting that cTFHs are not good markers of autoimmunity, allergy, or dysgammaglobulinemia in patients with DiGeorge syndrome.

### Expression of PD1 and ICOS Is Preserved on cTFHs of DiGeorge Syndrome Patients

**Figure 4:**
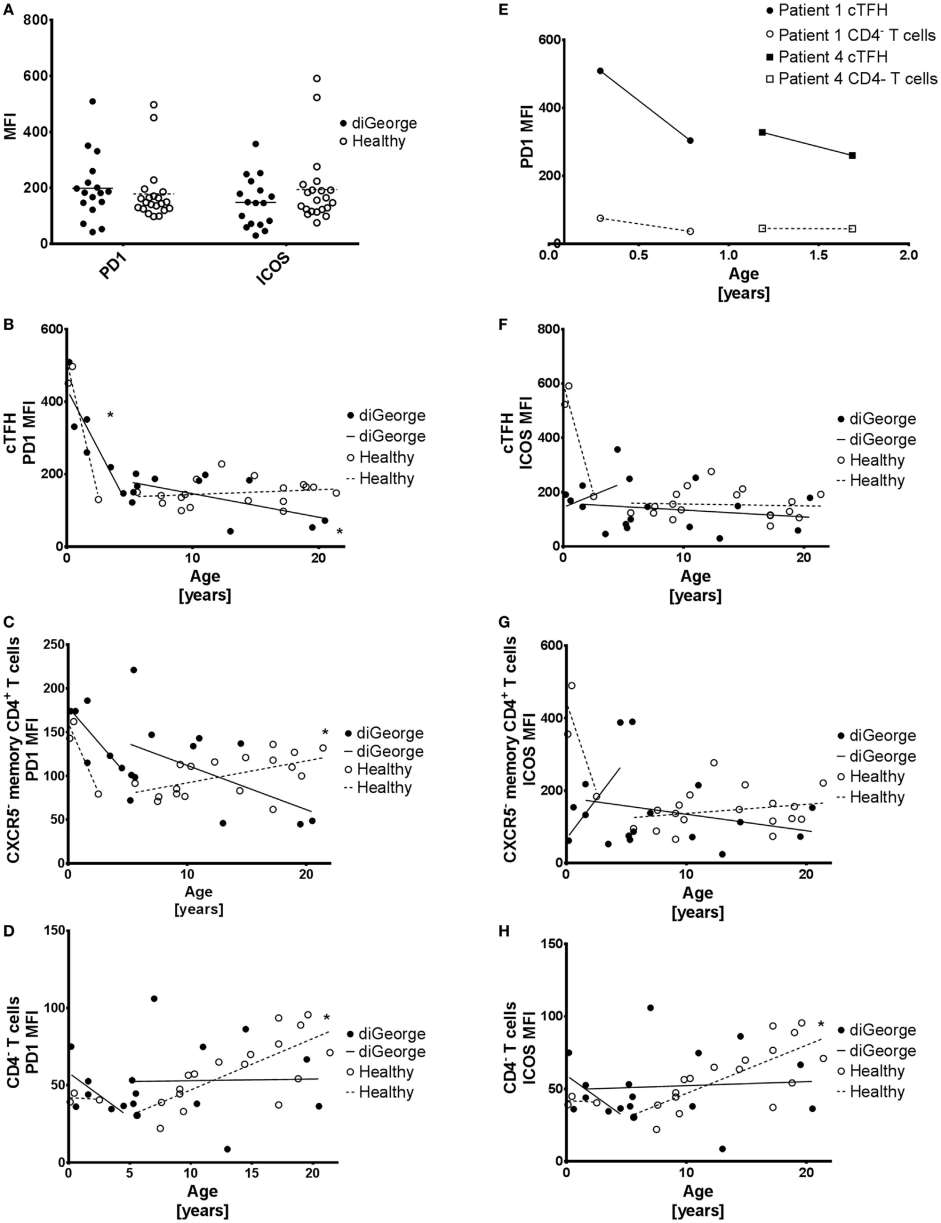
PD1 and ICOS expression on circulating follicular helper T cells (cTFH), CXCR5^−^ memory CD4^+^ T cells, and CD4^−^ T cells. **(A)** Summary graph showing PD1 and ICOS expression on cTFHs of DiGeorge patients and healthy controls. **(B)** Expression of PD1 on cTFHs, **(C)** CXCR5^−^ memory CD4^+^ T cells, and **(D)** CD4^−^ T cells with linear regression trendlines. * denotes significant correlation. **(E)** Change in PD1 expression over time on paired samples from two patients with DiGeorge syndrome on two consequent visits. **(F)** Expression of ICOS on cTFHs, **(G)** CXCR5^−^ memory CD4^+^ T cells, and **(H)** CD4^−^ T cells with linear regression trendlines. * denotes significant correlation.

The phenotypic and functional identity of cTFHs has been previously characterized using the extended surface expression of important surface molecules, such as the inhibitory checkpoint-molecule PD1 (26) and the costimulatory receptor ICOS (27, 28). We, therefore, evaluated the surface expression of PD1 and ICOS on cTFHs in DiGeorge patients compared to healthy controls, but saw no significant difference (multiple *t*-tests with Benjamini FDR approach, PD1 *p* = 0.57, ICOS *p* = 0.22) (Figure 4A).

### PD1 and ICOS Expression

Two healthy and three DiGeorge outliers with very high PD1 and ICOS expression on cTFHs can be seen in the summary data (Figure 4A). These are very young (<2 years old) patients and controls. Observing this trend, we evaluated the development of PD1 and ICOS expression in DiGeorge patients and healthy controls with age.

We observed a significant decrease of PD1 expression on cTFHs (linear regression, *p* = 0.01, *R*^2^ = 0.78) (Figure 4B), but not CXCR5^−^ memory CD4^+^ T cells (Figure 4C) or CD4^−^ T cells (Figure 4D) in the first 5 years of life in patients with DiGeorge syndrome. This trend seems to be similar in healthy controls, but is not significant, possibly due to low number of samples (*n* = 3) and, therefore, low statistical power.

PD1 expression further decreases later in life on cTFHs (linear regression, *p* = 0.04, *R*^2^ = 0.37) (Figure 4B), but not CXCR5^−^ memory CD4^+^ T cells (Figure 4C) and CD4^−^ T cells (Figure 4D) in DiGeorge syndrome. In healthy controls, there is no subsequent decrease in cTFHs, but on the contrary, there is significant increase of PD1 expression later in life on both CXCR5^−^ memory CD4^+^ T cells (linear regression, *p* = 0.01, *R*^2^ = 0.29) and CD4^−^ T cells (linear regression, *p* = 0.0003, *R*^2^ = 0.56), which is not present in DiGeorge patients.

There are no significant changes in ICOS expression on any measured T cell population of DiGeorge syndrome patients (Figures 4F–H). In healthy controls, however, there is a highly significant gradual increase of ICOS expression on CD4^−^ T cells in children older than 5 years. There seems to be a trend similar to the sharp PD1 decrease in first 5 years of life on cTFHs and CXCR5^−^ memory CD4^+^ T cells in healthy controls, but it is not significant.

We verified the observed strong decline of PD1 expression on cTFHs cells during the first 5 years of life on paired samples obtained from two DiGeorge syndrome patients on two consequent visits (Figure 4E).

## Discussion

We explore in this manuscript the presence and phenotype of TFH cells in the context of thymic pathology in patients with DiGeorge syndrome. As our understanding of the immune system grows more detailed, valuable opportunities present themselves at times to elucidate facets of long-known diseases that were not fully understood when those diseases were first described. One such opportunity was the description of the circulating population of follicular helper-like T-cells, which express the chemokine receptor CXCR5 and show functionally and transcriptionally distinct properties.

The inability of immune system in DiGeorge syndrome to produce naïve T-cells in normal quantities has been recognized for a long time and is believed to result from thymic dysplasia. We corroborate these findings on our cohort of pediatric DiGeorge syndrome patients, showing that there is a relative, but not absolute expansion of mature T-cells.

Circulating follicular helper T cells have already been studied in several primary immunodeficiencies, especially with emphasis on T cell B cell cooperation. For example, patients with hyper-IgM syndrome due to CD40L deficiency had low cTFHs (23, 29). The importance of B cells for cTFH generation was also shown in patients with BTK deficiency, who also had low cTFHs (30). Finally, the influence of cTFH on immune system dysregulation was also shown in CVID patients, who suffer from impaired antibody production and low-switched memory B cells, and in whom, elevated Th1 subset of cTFHs was associated with complicated course of the disease (24).

However, we show here elevated cTFHs in patients with thymic pathology as part of the DiGeorge syndrome, a finding previously heralded by Derfalvi et al. who also observed elevated bulk and activated cTFHs in DiGeorge syndrome patients (25). Similar trend of elevated cTFH counts was also shown in CVID patients with low-switched memory B-cells (31), and in the context of autoimmune disease such as systemic lupus erythematosus (32), Sjögren’s syndrome (33), or rheumatoid arthritis (34). Patients with DiGeorge syndrome also exhibit low class-switched memory B cells as we have shown previously (14); however, in our cohort, we saw no correlation between switched memory B cells and cTFHs, which was also not observed by Derfalvi. While we have not observed any difference in cTFHs between patients with and without history of autoimmune thrombocytopenia, the size of our cohort and lack of patients with other autoimmune complications precludes far-reaching conclusions at this point.

While there have been reports of hypogammaglobulinemia in patients with DiGeorge syndrome (12, 13, 35), which would be expected due to the lack of switched memory B cells, we have shown previously and again in this manuscript that there is a trend toward humoral immune dysregulation and hypergammaglobulinemia in DiGeorge syndrome, especially in adolescents. Although the increased numbers of cTFHs present one possible explanation, we found no correlation between serum IgG levels and cTFH numbers. Such correlation has been shown in the literature for some specific diseases such as IgG4-related disease (36) or rheumatoid arthritis (37), but is otherwise not widely observed, which might reflect the more complex nature of antibody production regulation.

We thus propose that cTFHs are present in patients with DiGeorge syndrome but are dysfunctional in their control and regulation of germinal center response, a hypothesis supported by the observed hypergammaglobulinemia and increased prevalence of autoimmune complications in DiGeorge syndrome and also in our cohort. Thymic dysplasia with loss of central tolerance may lead to production of autoreactive cTFHs resulting in autoimmune complications. Homeostatic proliferation of naïve T cells early in life that has been shown in DiGeorge syndrome (38) may also contribute to the relative expansion of cTFHs, a hypothesis supported by our finding of gradual increase of CXCR5 expression in healthy, but not DiGeorge cTFHs over time, which would indicate early expansion, but impaired long-term maturation of the cTFH compartment.

Finally, we provide data on the changes of PD1 and ICOS expression on various T cell subsets, including cTFH, over time. PD1 is a molecule that has enjoyed a dramatic increase in popularity in recent years, with the advent of checkpoint-blockade treatments in various cancers and with central role in CD8 T-cell exhaustion investigated primarily in chronic viral infections. While much has been documented about the expression of PD1 on cells in adults, there is little to no information available on its expression pattern during childhood. We observed a strongly increased expression of PD1 on cTFHs, and to lesser extent also CXCR5^−^ memory CD4^+^ T cells, in infants in both DiGeorge syndrome patients and healthy controls. We then show a much slower and gradual increase of PD1 expression in CXCR5^−^ memory CD4^+^ T cells and CD4^−^ T cells in healthy controls, but not DiGeorge syndrome patients, in older childhood and adolescence.

The trend of gradual PD1 expression increase has also been observed in murine CD4^+^ T cells (39) and CD8^+^ T cells (40). High levels of PD1 have also been reported in human neonatal Vdelta2 T cells (41), as well as cord blood Tregs (42). The exact impact of PD1 expression on the function of cTFHs is unclear, however, with both increased (21) and decreased (21) humoral immune response recorded in models with attenuated PD1/PD1L interaction. Interestingly, in their study Derfalvi et al. show increased percentage of CXCR5^+^CCR7^lo^PD1^hi^ activated cTFH CD4 T cells in both pediatric and adult DiGeorge syndrome patients. Considering the fact that we did not observe increased PD1 expression on DiGeorge cTFHs compared to controls, this finding by Derfalvi et al. may be attributed to higher proportion of bulk cTFHs in CD4 T cells.

In summary, we present here novel information on cTFHs in patients with thymic pathology and primary immunodeficiency underlying DiGeorge syndrome and provide first data on the change in PD1 and ICOS expression on cTFHs and other T cell subsets during childhood. Our work proposes new challenges for investigation in patients with primary immunodeficiency, which could lead to better understanding of the function of cTFHs, as well as temporal development of PD1 and ICOS expression.

## Ethics Statement

This study was carried out in accordance with the recommendations of the Ethical Committee of the second Faculty of Medicine, Charles University in Prague and University Hospital in Motol, Czech republic. The protocol was approved by the Ethical Committee. All subjects gave written informed consent in accordance with the Declaration of Helsinki.

## Author Contributions

AK designed the study, performed experiments, analyzed results, and wrote the manuscript. ZP designed the study and performed the experiments. MB and JP assisted with sample acquisition and preparation and provided clinical information. MR performed experiments. SU analyzed the data, interpreted results, and contributed to the discussion. KW contributed to the discussion of data and writing of the manuscript. AŠ interpreted results, assisted with sample acquisition and preparation, and provided clinical information and co-wrote the manuscript. All authors contributed to manuscript revision, read and approved the submitted version.

## Conflict of Interest Statement

The authors declare that the research was conducted in the absence of any commercial or financial relationships that could be construed as a potential conflict of interest.
